# Improving sleep in ICUs through real-time sleep monitoring: a proof-of-concept study

**DOI:** 10.1016/j.aicoj.2026.100059

**Published:** 2026-04-01

**Authors:** Xavier Drouot, Quentin Heraud, Marie-Anne Melone, Stéphanie Ragot, Jean Pierre Frat, Remi Coudroy, Florence Boissier, Anne Veinstein, Delphine Chatellier, François Arrivé, Sylvain Le Pape, Laura Marchasson, Christophe Rault, Arnaud W. Thille

**Affiliations:** a INSERM, CIC 1402, Equipe IS-Alive, Université de Poitiers, UFR de Santé, Poitiers, France; b CHU de Poitiers, Service de Neurophysiologie Clinique, Poitiers, France; c INSERM U-1084, Laboratoire de Neuroscience Expérimentales et Cliniques, Poitiers, France; d CHU de Rouen, Service de Pneumologie, Oncologie Thoracique et Soins Intensifs Respiratoires, Rouen, France; e CHU de Poitiers, Service de Médecine Intensive et Réanimation, Poitiers, France; f CHU de Poitiers, Service d’Explorations Fonctionnelles, Physiologie Respiratoire et de l’Exercice, Poitiers, France

**Keywords:** Sleep deprivation, Intensive care unit, Sleep monitoring, Sleep quantification, Nurse care, Sleep promotion

## Abstract

**Background:**

Sleep disturbances are associated with a poor prognosis in intensive care units (ICUs). Nursing care during the night can further disturb patient sleep. We hypothesized that nursing rounds and care would be less harmful to sleep if guided by a real-time sleep monitoring system.

**Methods:**

This was a quasi-experimental, prospective, before-after study including non-sedated ICU patients. In the usual care group (first group recruited), nursing rounds and care were performed every four hours as daily practice, regardless of the patient’s sleep/wake status. In the sleep-guided care group (second group recruited), nursing rounds and care were guided by a sleep-EEG monitoring system indicating the patient’s sleep status in real time on a tablet positioned at the entrance of the room. When patients fell asleep, the tablet displayed a symbol asking caregivers to postpone non-urgent care. Otherwise (patient awake), nursing care was encouraged. The number of room entries was measured using an entry-exit counter, synchronized with sleep-EEG recorder. The system stored EEG in both groups. Two experts, blind to the patients’ groups, provided consensual sleep scoring. Primary outcome was continuous sleep (i.e. time spent in long sleep episodes).

**Results:**

Forty-six patients mainly admitted for acute respiratory failure (72% of cases) were analysed. Patients’ characteristics did not significantly differ between groups. The proportion of room entries while patients were asleep decreased from 22% [4–32] in usual care group to 6% [0−13] in sleep-guided care group (p = 0.015). Continuous sleep was longer in the sleep-guided care group (20 patients) compared to the usual care group (26 patients): 170 min [75–240] vs. 80 min [53–128] (p = 0.03). Deep sleep was likewise longer in the sleep-guided care group.

**Conclusion:**

Our study reports for the first time that real-time sleep monitoring can guide nursing care and improve sleep quality in ICUs.

## Highlights

• Sleep alterations are frequent in ICU and associated with poor outcomes.
• Nursing care during the night frequently disturb sleep.
• Sleep monitoring system can inform caregivers when patients are asleep and awake.
• Using a sleep monitoring system allows caregivers to postpone non urgent care.
• Sleep monitoring system increases sleep quality and quantity.

## Introduction

Sleep is a critical function for human beings and sleep loss causes behavioural abnormalities such as fatigue, cognitive and motivational deficits [1], dramatic reduction in inspiratory endurance [2], abnormal glucose control [3] and altered immune functions [4].

Sleep organization is severely disrupted in patients admitted to intensive care units (ICUs) [5,6], where critically-ill patients spend most of their sleep in numerous very short sleep episodes, usually lasting 4−10 min [7]. Sleep episodes of longer duration (more than 10 min) are restorative but uncommon [7]. This sleep pattern results in severe sleep deprivation.

Several studies have reported an association between sleep alterations and poor ICU outcomes [6]. In patients with hypercapnic acute respiratory failure, sleep alterations have been associated with a higher risk of intubation [8]. More recently, several studies have reported that non-sedated intubated patients with severe sleep disruptions go through weaning phases of longer duration [[9], [10], [11]]. Some authors have also reported an association between sleep disruptions and higher mortality rate [[12], [13], [14]] and some studies have suggested that sleep disruptions could lead to delirium [15].

Reasons for poor sleep in ICU comprise individual factors, such as anxiety, premorbid insomnia and high levels of environmental noise [16]. Nighttime care activities occur frequently, up to 51 times per night [17,18] and often cause awakenings and sleep interruptions [19]. Consequently, ICU care-related activities have been reported as one of the most sleep disruptive factors by patients [20].

Consequently, a consensus has emerged that improving sleep may rely on the implementation of bundles of care, including reducing and clustering nighttime nursing care. However, these interventions have yielded disappointing results [[21], [22], [23], [24], [25]].

We hypothesized that if nurse rounds and care were guided by a system indicating in real time the sleep or wake status of the patient, short nascent sleep episodes could be transformed into long restorative sleep episodes. We expected sleep-guided nurse care to increase sleep quality.

## Methods

### Study design and patients

This prospective, single-center quasi-experimental “before-after” study was conducted at the Medical ICU of the University Hospital of Poitiers in France. It was approved on March 27th, 2023 by the national ethics committee (Comité de Protection des Personnes, CPP Ile de France II, number #2022-A01806−37) and registered at http://www.clinicaltrials.gov (NCT05963672). Patients and/or their next of kin were informed and gave written consent before being included in the study. All patients admitted to the ICU, conscious (RASS >−2 and <1) and free from sedatives for at least 24 h were eligible for inclusion. Inclusion criteria were: (a) adults admitted to the medical ICU of the University Hospital of Poitiers; (b) patients who had been intubated for more than 24 h or were not intubated; (c) patients free of sedation for more than 24 h with a Richmond Agitation–Sedation Scale (RASS) score between −2 and +1; and (d) patients who gave his/her written consent. Patients suffering from cerebral disease (tumour, epilepsy, stroke, neurodegenerative disease) and patient receiving drug known to interfere with sleep or sleep EEG (benzodiazepine, sedatives, hydroxyzine, antidepressant, antipsychotics and melatonin) were not included in the study.

**Fig. 1:**
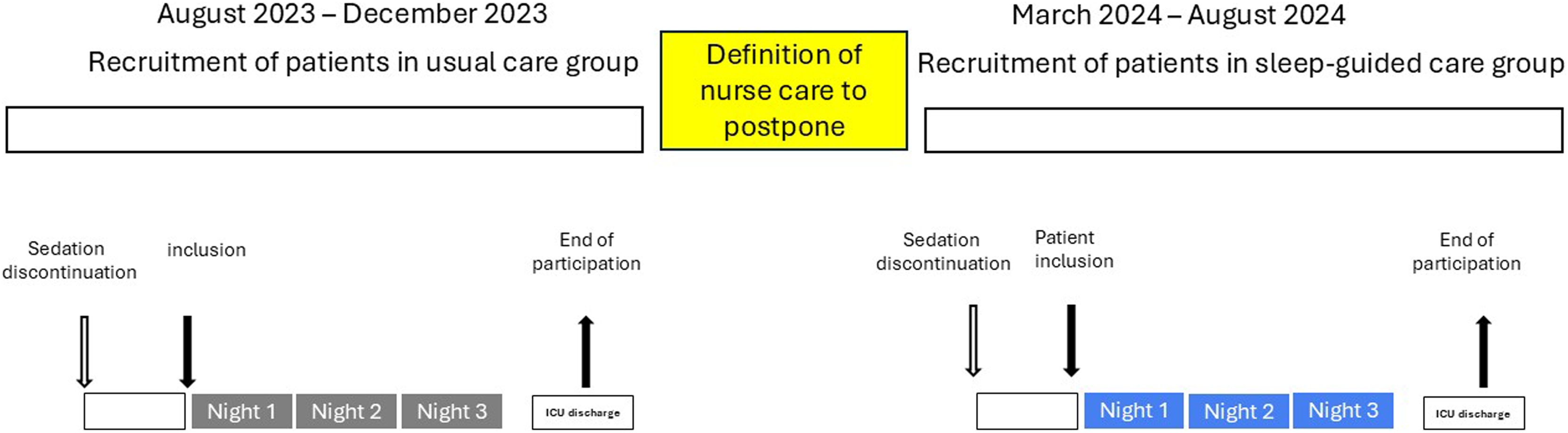
Study design.

We first recruited a control group receiving usual care. We secondly elaborated the sleep-guided nursing care procedure. Finally, we recruited the sleep-guided nursing care group who were to receive sleep-guided nursing care (Fig. 1).

### Study intervention

Sleep was monitored using a micro sleep-EEG monitor (Sleepscan°, Somnoengineering Company, Poitiers, France, see below) from 7 p.m. to 7 a.m. for a maximum of three consecutive nights, whenever possible.

In the usual care group, sleep was recorded but caregivers were blind to patient sleep status (the tablet indicating patient sleep state was hidden). Nursing care and nurse rounds were performed as usual at least every four hours or more, according to the patient’s severity, without specific regard to their sleep status.

In the sleep-guided nursing care group, sleep was recorded and all caregivers were aware of a patient’s sleep status by means of a tablet located at the entrance of the patient’s room, indicating that patient’s sleep state (awake or asleep) in real time.

Study intervention consisted in nursing care performed according to the patient’s sleep status (i.e. sleep-guided nursing care). Procedures for sleep-guided nurse care were elaborated using a modified DELPHI method with nurse team. When the system indicated that patient was sleeping, caregivers had to refrain from entering the room and to postpone nurse rounds and all non-urgent care. Otherwise, when system indicated that patient was awake, caregivers were encouraged to anticipate and perform all necessary nursing tasks. As recommended by recent guidelines [5], the intervention was applied during nighttime only and not during the daytime, as promoting daytime sleep could exacerbate circadian rhythm abnormalities.

The following adverse events were monitored by the intensivist in charge of the patient: skin irritation under the electrodes, a desire to withdraw the study, a worsening of neurological status of the patient (defined by an increase of 1 point on RASS score at 8am) and discomfort during ICU stay assessed using IPREA scale (Inconforts Perçu par les patients en REAnimation). In addition, all serious adverse events (death, worsening of patient’s condition, a new disease or injury compromising patient’s outcome, a new organ failure, a prolonged ICU stay or need for a surgical intervention) were monitored independently by the hospital’s medical device vigilance department.

### Real-time sleep monitoring medical device

**Fig. 2:**
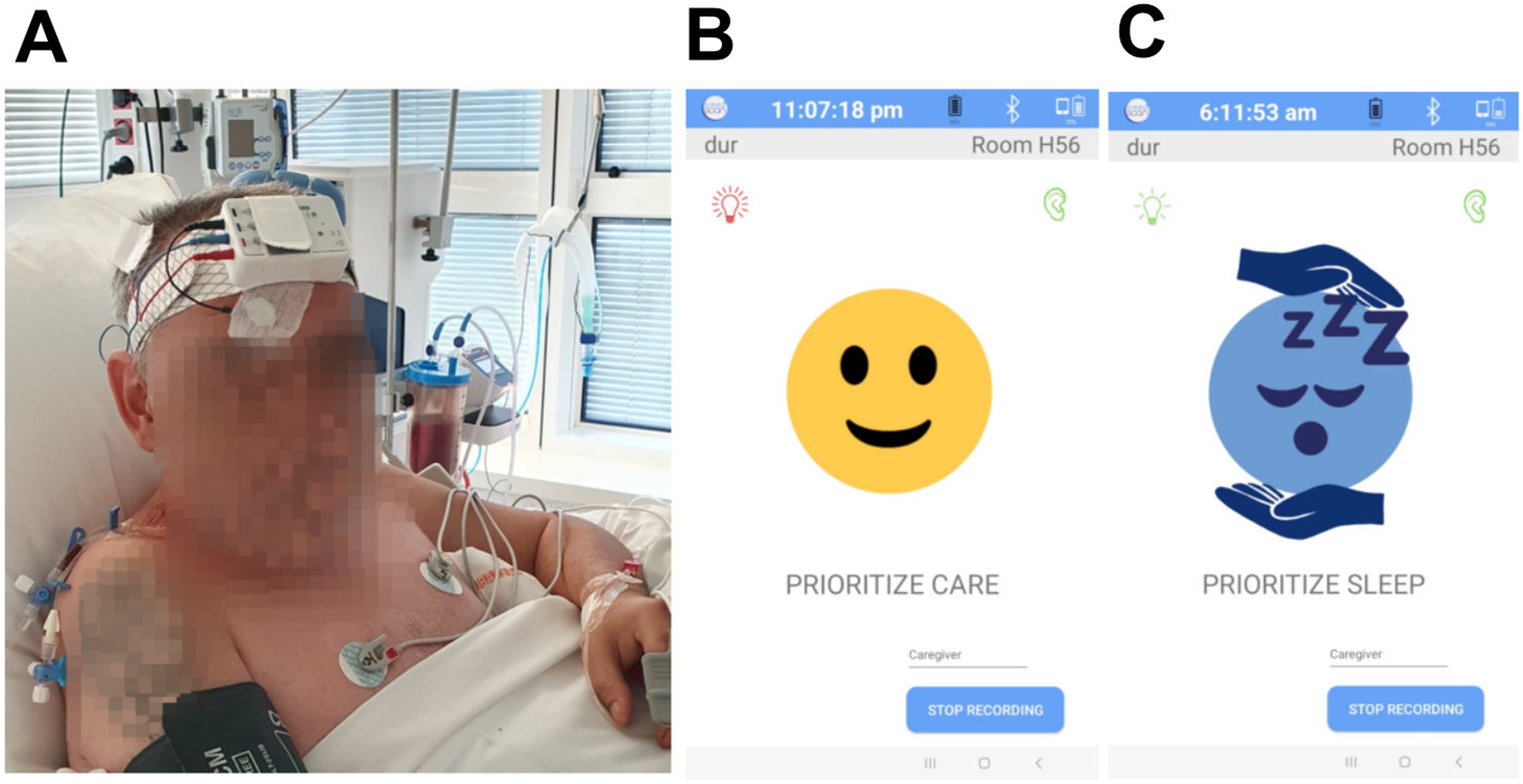
Sleep monitoring system. A: Sleepscan medical device; B: Picture displayed by the tablet when patient is awake; C: Picture displayed by the tablet when patient is asleep.

This system is composed by a micro-sleep-EEG monitor recording a single EEG channel (C4-M1 or C3-M2) embedding an automated sleep scoring algorithm. This algorithm detects all conventional American Association Sleep Medicine sleep stages as well as atypical sleep and pathological wakefulness. It has been validated against visual scoring on polysomnography recordings [26] in non-sedated ICU patients; the algorithm detects 99% of sleep episodes lasting more than 10 min. The monitor is connected wirelessly to a tablet displaying the results of the scoring at the doorstep of patient’s room. The system informed care givers as follows: When the patient was asleep for more than 10 min, the tablet displayed a blue sleeping smiley. When the patient was awake, the tablet displayed a yellow awake smiley (Fig. 2).

### Sleep recording and analysis

The device stored the raw EEG signal. All EEG recordings lasting more than six hours with less than 50% artifacts could be analysed. We decided to analyse only nights with at least one sleep episode lasting more than 10 min for two reasons: First, sleep-guided nursing care are provided only if patient sleeps for at least 10 min. Second, the quantification of sleep effectiveness requires a minimal amount of sleep [27]. Two experts (MAM and XD), familiar with sleep EEG patterns in ICU patients and blind to the patients groups, provided consensual sleep scoring using conventional rules [28] or alternative rules for atypical sleep [29]. Sleep quantification included amount of non-rapid-eye-movement sleep stages 1, 2 and 3 (N1, N2, N3) and rapid-eye-movement (R) sleep, and presence of atypical sleep. Additionally, we quantified N3 sleep amount off-line by calculating the slow wave activity (SWA): SWA was defined as the relative power spectra of the delta band (1–4 Hz/1 Hz–30 Hz) and was calculated for all the epochs scored sleep by the automated algorithm. Then, the 75th value of SWA across sleep epochs was calculated for each recording and sleep epochs displaying a high SWA (SWA above the individual 75th centile) were defined as N3 sleep epochs [30]. Continuous sleep was defined as the amount (in min) of sleep spent in episodes lasting more than 10 min, whatever the sleep stage. Numerous studies have demonstrated that a period of at least 10 min of uninterrupted sleep is required for restorative processes to occur [31]. In ICU patients, a high proportion of sleep occurring in episodes lasting more than 10 min has been associated with favourable respiratory outcomes [7] and satisfactory sleep [27]. Although 10 min of sleep may have physiological relevance, this duration remains relatively short for ICU patients. We therefore also investigated whether the intervention increased the time spent in sleep episodes lasting more than 30 min, a threshold that has been previously validated [7,32].

Sleep efficiency was defined as the ratio of total sleep time divided by recording time. Sleep fragmentation was estimated by counting the number of arousals and awakenings per hour of sleep. An awakening is a shift from an epoch of sleep to an epoch of wake; an arousal is a brief irruption of wake (3−15 sec duration) in an epoch of sleep.

Each morning, the patient’s reported sleep quality was estimated using Richards Campbell Sleep Questionnaire (RCSQ), while anxiety was measured using Spielberger State-Trait Anxiety Inventory and presence of delirium was searched using Confusion Assessment Methods in ICU (CAM-ICU).

### Room entries

Room entries were counted and exact time of each entry was recorded using an entry-exit counter. To investigate the effect of room entries on sleep, the entry–exit counter was synchronised with the Sleepscan medical device. The timing of each room entry was then aligned with the sleep scoring. For each patient, we measured the median sleep amount during the 10 min before and the 10 min after each entry occurring while the patient was asleep. We then compared the average sleep amount before and after entries across patients in the usual care group and in the sleep-guided care group, separately.

### Environmental noise levels and nurse’s perceived workload assessment

Environmental noise (dBA) in the room was recorded by sound meter embedded in the micro sleep-EEG monitor and analysed offline. A noise peak was defined by an increase of sound level of at least 10 dB with a slope of at least 10 dBA/second.

The night nurse’s perceived workload caused by the device was estimated using a visual analogue scale going from 0 (“no additional workload”) to 10 (“considerable additional workload”).

### Outcomes

The primary outcome was sleep effectiveness defined by the amount of continuous sleep on the first analysable night. Secondary outcomes included total sleep time, sleep stage composition, sleep fragmentation, number of noise peaks >60 dB during sleep, and percentage of room entries while patients were asleep.

In addition, some measures were averaged across all study nights including the proportion of continuous sleep, patients’ reported sleep quality, night-nurse workload, patient anxiety level and number of patients with positive CAM-ICU.

### Statistical analysis

Continuous variables were expressed as median and interquartile [25th-75th percentiles] range (IQR), and qualitative variables were expressed as number and percentage. All variables were compared using the non-parametric Fisher’s exact test for categorical variables and the Mann-Whitney test for continuous variables. A two-tailed P-value of less than 0.05 was considered statistically significant. All analyses were performed using R statistical package.

Based on previous studies [11], we estimated that 24 analysable patients had to be included in each group in order to detect a difference of 120 min of continuous sleep between groups, with an alpha risk of 0.05 and a power of 0.8. Considering a 25% rate of recording failures, we decided to recruit 30 patients by groups. Furthermore, because this was the first use of this device, we anticipated that some recordings might be non-analysable. We therefore planned to enrol as many patients as necessary to obtain 30 patients with at least one analysable night.

## Results

### Patients

Eighty patients were included in the study: 44 were included in the usual care group (during the first period from August to December 2023), and 36 in the sleep-guided nursing care group (during the second period from March to August 2024). After excluding 18 patients (22%) for whom EEG analysis was not possible due to electrode displacement and two patients who met the exclusion criteria, 60 patients had recordings. Among them, 14 patients (4 in the usual care group and 10 in the sleep-guided care group) had no sleep episode longer than 10 min.

**Fig. 3:**
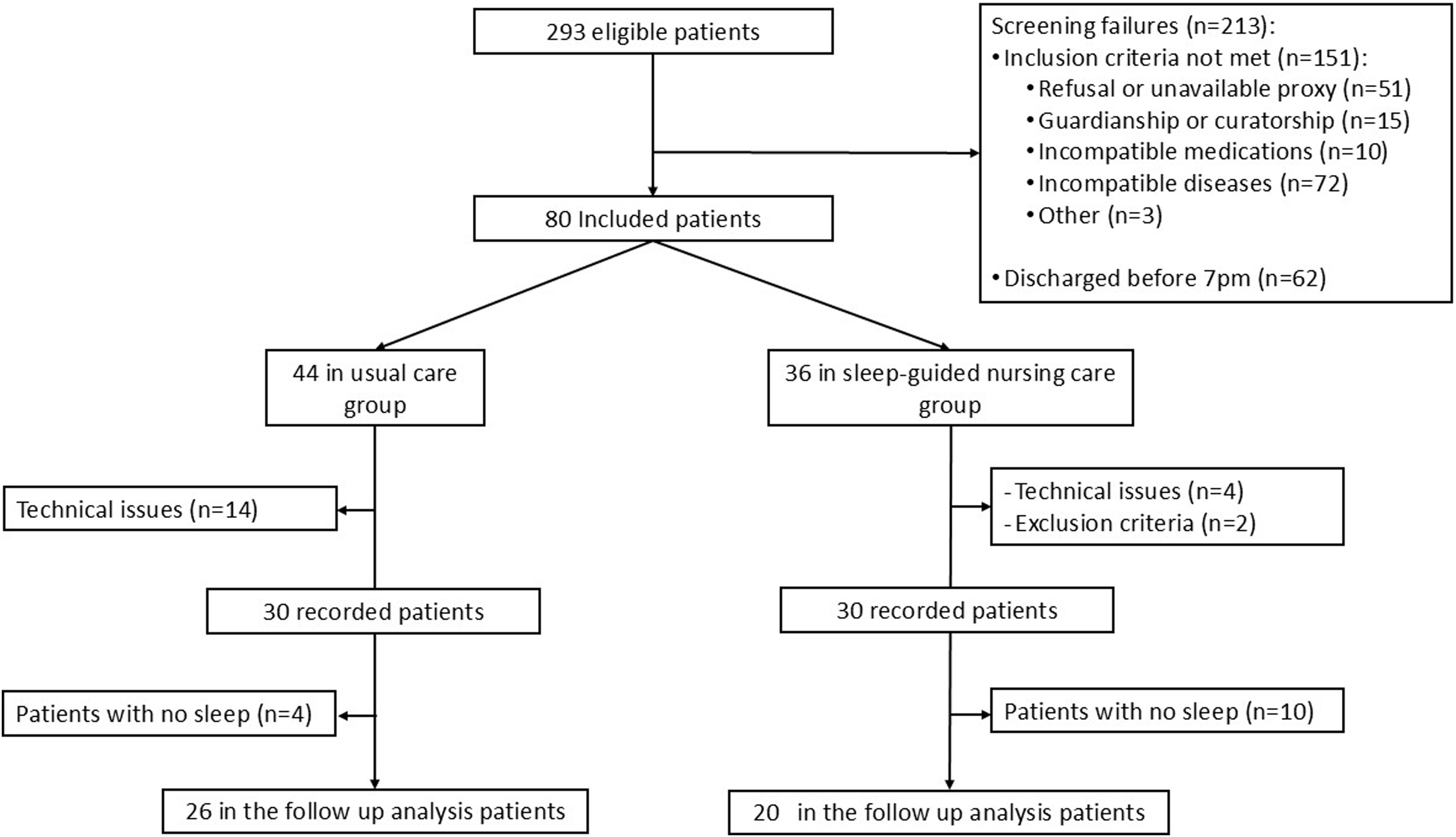
Flow chart of the study.

Finally, 46 patients were retained in the follow-up analysis, including 26 patients in the usual care group and 20 patients in the sleep-guided nursing care group (Fig. 3). The main reason for admission was acute respiratory failure in 33 patients (72%). Two patients (8%) in usual care group and one patient (5%) in sleep guided care group were mechanically ventilated. The median number of study nights was 2 [1-2] in usual care group and 1 [1-2] in sleep guided-care group (p = 0.28). Patients’ data regarding medical history before inclusion and patients’ characteristics at inclusion did not significantly differ between groups (Table 1 and Table 2). CAM-ICU scores at inclusion were available for 23 patients among 26 (88%) in usual care group and for 13 patients among 20 (65%) in sleep-guided care group. One patient among 23 (4%) had a positive CAM-ICU in usual care group; no patient among 20 had a positive CAM-ICU in sleep-guided nursing care group.

**Table 1:** Comparison of demographic and clinical data between the two groups.

	Usual care group	Sleep-guided care group	*p*
Patients (n)	26	20	
Age (y)	64 [56–67]	64 [46–71]	0.86
Male (n, %)	19 (73)	8 (40)	0.04
Body Mass index	26 [24–30]	28 [23–33]	0.67
Severity at admission (SAPS II)	39 [33–49]	34 [24–47]	0.31
Reasons for admission (n,%)			0.51
- Acute respiratory failure	18 (69)	15 (75)	
- Shock	3 (11)	3 (15)	
- Neurological reasons / coma	1 (4)	2 (1)	
- Post-surgery	1 (4)	0 (0)	
- Others	3 (11)	0 (0)	
Need for invasive mechanical ventilation before inclusion (n,%)	8 (31)	9 (45)	0.37
Median duration of sedation (days)	7.5 [2.5–14]	2.5 [1–6.3]	0.2
Propofol (cumulative doses, mg)	12,715 [7,374–44,047]	3,528 [1,667–8,963]	0.1
Midazolam (cumulative doses, mg)	0 [0–78]	0 [0 – 0]	0.1
Time from sedation discontinuation (days)	3.5 [1.8–4.3]	2.0 [1.0–4.0]	0.4
Time from admission (days)	2.0 [1.0–4.5]	2.5 [1.0–5.3]	0.8

Values are median [25th – 75th]. y: years; SAPS II: Simplified Acute Physiology Score.

**Table 2:** Comparison of clinical data at inclusion.

	Usual care group	Sleep-guided care group	*p*
Patients (n)	26	20	
Invasive mechanical ventilation (n, %)	2 (8)	1 (5)	1
PaO_2_/FIO_2_ (mmHg)	218 [198–289]	283 [176–312]	0.94
pH (units)	7.46 [7.40–7.48]	7.49 [7.44–7.51]	0.24
PaCO_2_ (mmHg)	35 [33–37]	39 [29–42]	0.94
SOFA score	2.5 [2–5]	2.5 [2–6]	0.9
RASS score	0 [0 – 0]	0 [0 – 0]	1
Glasgow score	15 [15–15]	15 [15–15]	0.8
Patients with available CAM-ICU (n)	23	13	
Delirium, positive CAM-ICU n (%)	1 (4)	0 (0)	1

Values are median [25th – 75th]; PaO2: oxygen arterial partial pressure; FIO2: Inspired fraction of oxygen; PaCO2: CO_2_ arterial partial pressure. SOFA: Sequential Organ Failure Assessment score; RASS: Richmond Agitation Assessment Score; CAM-ICU: Confusion assessment Method for the ICU.

### Intervention: reorganisation of room entries

Number of room entries per night was similar in the usual care group and the sleep-guided nursing care group (26 [20–32] vs. 23 [16–31] respectively; p = 0.4). The proportion of room entries occurring while patients were asleep was lower in the sleep-guided nursing care group than in the usual care group (6% [0−13] vs. 22% [4,21–32] respectively, p = 0.015) (Table 2).

**Fig. 4:**
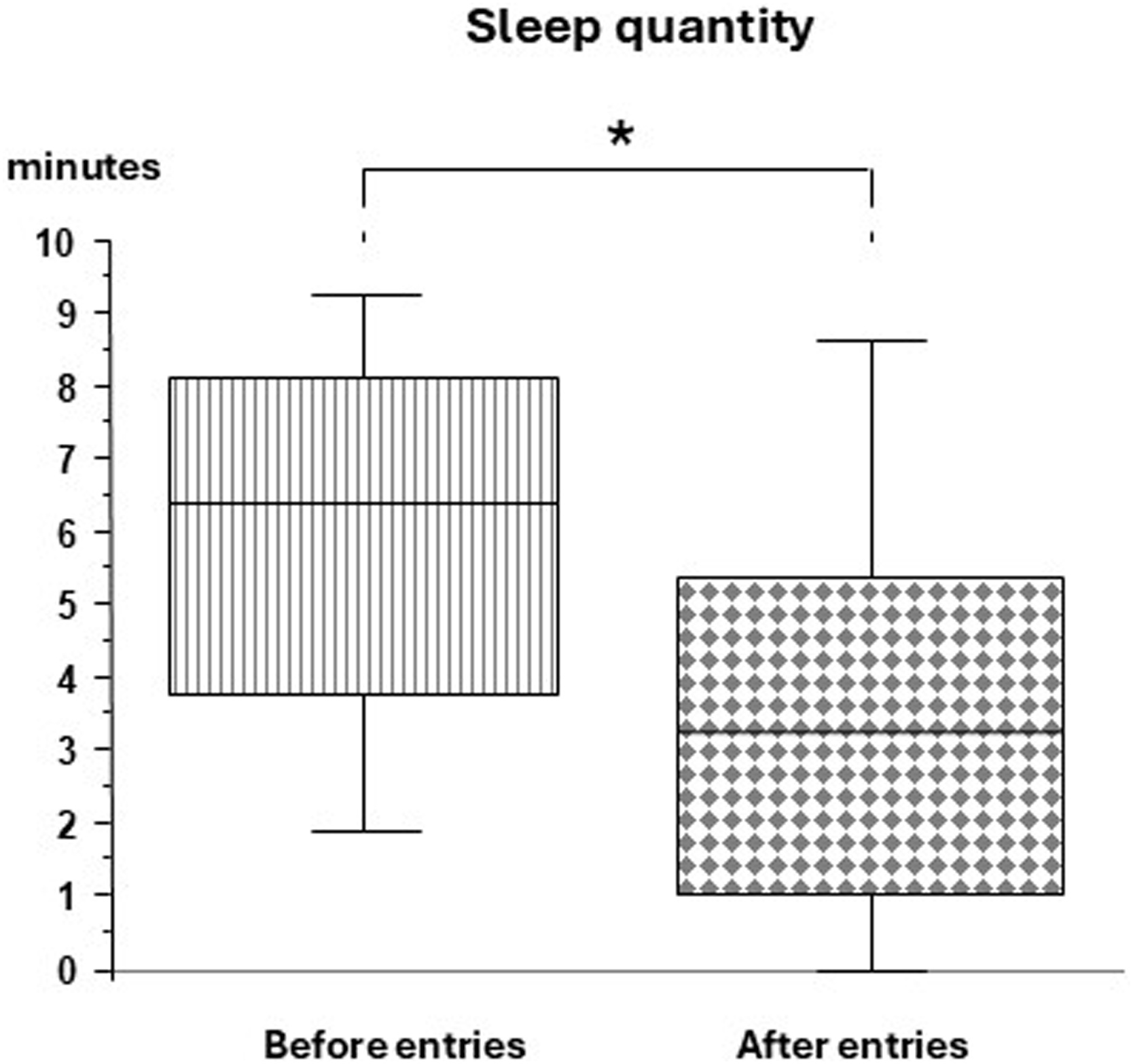
Sleep quantity before and after room entries in usual care group. Box graph illustrating the 25th quartile (bottom line of the box), the median (middle line), the 75th quartile (upper line of the box), the 10th centile (bottom whisker) and the 90th centile (upper whisker) of sleep quantity before entries (left box, vertical stripes; n = 26) and after entries (right box, black squares, n = 26). *: *p* < 0.05 (Wilcoxon Signed rank Test).

The median amount of sleep *after* entries was significantly lower than *before* entries (3.3 min [1.0–5.3] vs 6.4 min [3.9–7.0], p = 0.01, respectively) in the usual care group (Fig. 4), whereas no significant difference was observed in sleep-guided care group (4.5 min [1.3–7.0] vs 6.0 min [4.3–8.4], p = 0.07, respectively).

No adverse events were reported in either group. No patient withdrew consent during the study.

### Sleep analysis

**Fig. 5:**
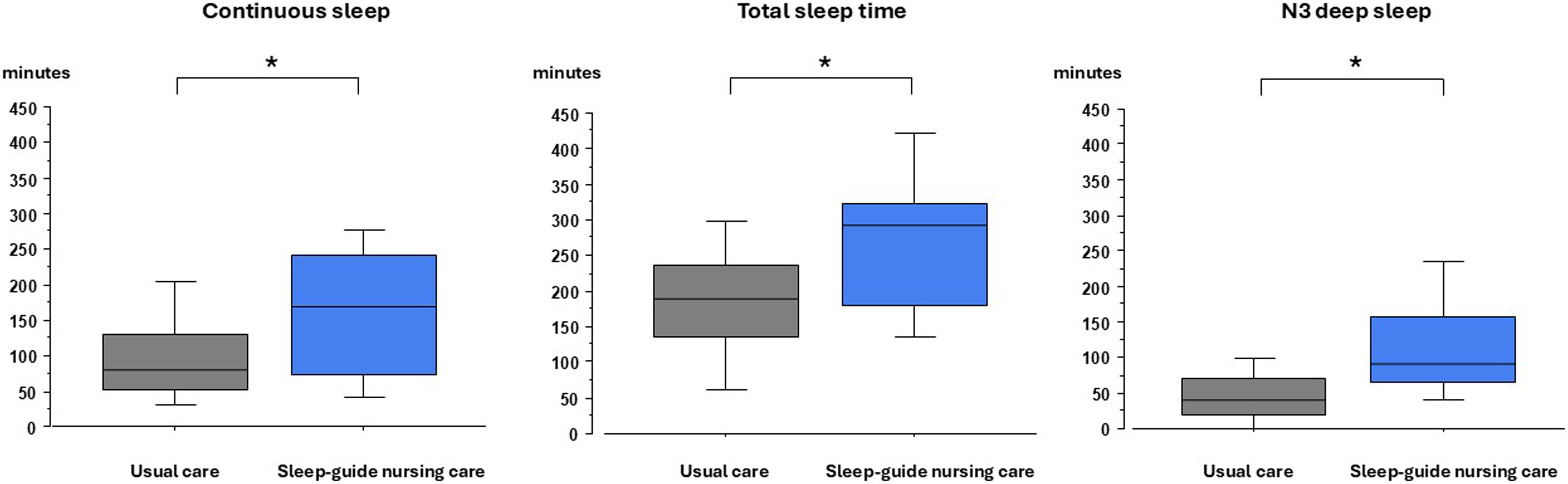
Continuous sleep, total sleep time and N3 deep sleep calculated on first night in the usual care group and in the sleep-guided nursing care group. All graphs illustrate sleep measured by blind and consensual visual scoring. Box graph illustrating the 25th quartile (bottom line of the box), the median (middle line), the 75th quartile (upper line of the box), the 10th centile (bottom whisker) and the 90th centile (upper whisker) for usual care group (left gray box, n = 26) and for sleep-guided nursing care group (right blue box, n = 20). *: *p* < 0.05 (Mann-Whitney test).

Sleep effectiveness assessed using continuous sleep on the first study night was higher in the sleep-guided nursing care group compared to the usual care group: 170 min [75–240] vs. 80 min [53–128] (p = 0.01) as well as sleep effectiveness (% of total recording time): (28% [13–42] vs 13% [9–21], p = 0.03) (Fig. 5). Sleep time spent in long episodes (>30 min) was higher in sleep-guided care group compared to usual care group (44 min [0–167] vs 34 min [0–82], p = 0.03, respectively). Sleep quantity as well as deep sleep (N3 sleep, assessed using blind visual scoring or automated scoring) were higher in the sleep-guided nursing care group compared to the usual care group (Table 3). Number of awakenings and arousals per hour of sleep, and number of noise peaks during sleep were similar in the two groups.

**Table 3:** Outcomes and sleep characteristics.

	Usual care group	Sleep-guided care group	*p*
Patient (n)	26	20	
**Primary outcome**			
Continuous sleep during the first night (min)	80 [53–128]	170 [75–240]	0.03
Continuous sleep during the first night (%TRT)	13 [9–21]	28 [13–42]	0.01
**Secondary outcomes**			
Sleep characteristics during the first night			
Room entries (total number)	26 [20–32]	23 [16–31]	0.40
Room entries while asleep (%)	22 [4–32]	6 [0−13]	0.02
Total recording time (min)	606 [594–630]	588 [558–606]	0.01
Total sleep time (min)	189 [136–235]	285 [205–323]	0.01
Sleep efficiency (%)	30 [21–39]	48 [39–58]	0.03
N1 (min)	23 [9–37]	21 [14–25]	0.45
N2 (min)	89 [49–122]	84 [67–150]	0.58
N3 (min)	38 [18–68]	93 [67–152]	<0.01
N3 (min) (automated scoring)	40 [15–100]	101 [43–154]	0.03
REM sleep (% of TST)	0 [0–11]	3 [0–31]	0.35
Arousals + awakenings (n/h)	32 [24–42]	21 [19–33]	0.11
Noise peaks during sleep (n/h of sleep)	190 [64–2,143]	720 [245–2,313]	0.20
Sleep characteristics during all study nights			
Number of study nights	2 [1–2]	1 [1–2]	0.28
Continuous sleep per night (% TRT)	13 [9–21]	29 [13–42]	0.01
Total recording time per night (min)	604 [588–621]	582 [549–611]	0.04
Total sleep time per night (min)	192 [123–234]	274 [179–333]	0.03
Sleep efficiency (% of total sleep time)	32 [20–38]	45 [33−58]	<0.01
Perceived sleep quality (RCSQ; n = 18; 16)	59 [47–77]	57 [48–69]	0.96
Anxiety score (Spielberger State Anxiety Inventory,(n = 12; 8)	29 [26–40]	26 [24–31]	0.18
Patients discomfort scale (IPREA score) (n = 16; 11)	20 [13–34]	12 [5–25]	0.11
Nurse perceived workload (0–10 scale)	1.0 [1.0–1.1]	1.4 [1.2–2.2]	0.02
Patients with positive CAM-ICU (n)	4	0	

Values are median [25th – 75th]. TRT: total recording time; N1: NREM sleep stage 1; N2: NREM sleep stage 2; N3: NREM sleep stage 3. R: Rapid-Eye Movement sleep; TST: total sleep time; RCSQ: Richards-Campbell Sleep Questionnaire; IPREA: Inconforts des Patients de REAnimation (discomfort for ICU patients); dBA: decibel A; n/h: number per hour of sleep; min: minute; The Spielberger State Anxiety Inventory score goes from 20 (low anxiety) to 80 (high anxiety). The Richards-Campbell Sleep Questionnaire goes from 0 (very poor sleep) to 100 (very good sleep). IPREA score goes from 0 (no discomfort) to 100 (very high discomfort). CAM-ICU: Confusion Assessment Method for ICU.

When averaging all study nights, amounts of continuous sleep and total sleep were higher in the sleep-guided nursing care group compared to the usual care group (Table 3).

Sleep quality reported by patients assessed using RCSQ did not significantly differ between groups. Anxiety score did not differ between groups. Although the score at the discomfort scale was higher in the usual care group, it did not reach the threshold of significance.

### Nurse perceived workload

Nurse perceived workload was significantly higher in the sleep-guided nursing care group (Table 3).

## Discussion

Our results report for the first time that when caregivers are aware of patients’ sleep status due to a real-time sleep monitoring system, they can modify their rounds and nursing care and limit room entries while patients are sleeping. Sleep-guided nursing care increases sleep continuity, sleep episodes lasting more than 30 min and deep N3 sleep. Real-time sleep monitoring paves the way for personalized and efficient non-pharmacological improvement of sleep quality and quantity in ICUs.

### Effects of sleep-guided nursing care on sleep effectiveness

To our knowledge, we show for the first time that sleep-guided nursing care using a real-time sleep monitoring system dramatically improves sleep effectiveness by increasing time spent in long sleep episodes. Our results extend the findings of several studies reporting improved sleep effectiveness after organization of nighttime quiet times [33,34]. However, these studies assessed sleep using subjective scales, which might be contaminated by perception bias [27]. As a strength, our study shows that sleep-guided nursing care had a direct beneficial effect on objective sleep effectiveness assessed using EEG recordings. In addition, both visual scoring and automated analysis showed increased N3 sleep amount with a trend toward restoration of a normal amount of N3 sleep [35]. Increased N3 sleep in the sleep-guided nursing care group was likely due to the longer sleep episodes induced by fewer sleep interruptions by nursing care [19]. Another explanation could be that sleep-guided nursing care offers patients an opportunity to sleep according to their preferred sleep schedules [5]. Indeed, the dramatic doubling of N3 sleep induced by sleep-guided nursing care was superior to the effect obtained by earplugs and eye masks in a similar patient group studied using polysomnography [36].

REM sleep duration was not increased in the sleep-guided care group. REM sleep is known to be strongly influenced by environmental factors [16] and by patients’ anxiety [37]. As these two factors were similar in both groups, they likely affected REM sleep in a similar manner. Furthermore, severe sleep deprivation is known to generate a strong slow-wave sleep pressure [38]. In patients receiving sleep-guided nursing care, this increased slow-wave sleep pressure may have preferentially enhanced slow-wave sleep duration rather than REM sleep. Of note, breathing during REM sleep is characterized by exclusive diaphragmatic functioning, with complete inhibition of inspiratory accessory muscles, making it an inherently vulnerable physiological state for breathing. A reduction in REM sleep duration might therefore represent an adaptation (or even defence) mechanism in response to diaphragm dysfunction accompanying respiratory failure [39]. The potential benefits of restoring a normal amount of REM sleep on breathing required further investigation in patients with acute respiratory failure.

Nevertheless, current literature has established the severe consequences of sleep loss on ICU outcomes [5,6]. Since sleep continuity and deep sleep are major prerequisites for sleep to be restorative, the better sleep induced by sleep-guided nursing care might reduce need for intubation [40], weaning duration [11], delirium incidence [40], length of stay in ICU [40] and to ameliorate patient experience in ICUs.

### Entries in sleeping patients’ rooms are reduced when sleep is monitored

The significant reduction of sleep quantity after entries compared to before entries in usual care group confirms that entries while asleep interrupt sleep, thereby demonstrating that usual nursing care are serious sleep disruptor [19].

The significantly reduced proportion of room entries in asleep patients (6%) receiving sleep-guided nursing care compared to usual care (22%) indicates that real-time instructions were strongly followed by caregivers. This lower proportion of room entries in asleep patients explains the higher amount of sleep in episodes lasting more than 30 min. Knauert and collaborators reported a similar reduction in room entries during night-time quiet time, but with real difficulties in implementing quiet-time procedures for more than two hours [41]. Displaying the patient’s sleep status at the doorstep in real time might considerably increase the opportunities for caregivers to let patients sleep. In contrast with several studies reporting no impact of quiet time on nurse workflow [42,43], nurse perceived workload was slightly higher in sleep-guided nursing care. Of note, the workload assessed was perceived rather than actual workload. The observed increase was therefore not unexpected, as additional organisational constraints may increase perceived workload. However, the amount of additional workload was very low (from 1 to 1.4 on a 0–10 scale) and no nurses requested to withdraw from the study.

Although not significant, noise peaks were more frequent in the sleep-guided group, despite less entries. Most of noise peaks are mainly alarms from monitors or ventilators [16] and were not targeted directly by our intervention.

### Limits of the study

The main limit of our study is that allocation of sleep-guided nursing care was not randomized, so that a confusion factor could explain the effect we observed. For example, in these mainly non-intubated patients, obstructive sleep apnea syndrome might have been more frequent in the usual care group, resulting in poorer sleep effectiveness in this group. Considering the difficulties in implementing a similar protocol [44], we wished to increase the efficiency of the procedure and the motivation of the nursing team by temporally separating the two arms in a “before – after” designed study. Other confounders such as changes in nursing practice, medication use, pain management or nocturnal procedures might have evolved between study periods and might have influence sleep outcomes.

A second limit is that the recruitment period differed according to the season, with the control group mainly recruited in fall and winter, and the sleep-guided care group recruited in spring and summer. All rooms had windows with natural light exposure. Knowing the impact of natural light on sleep [45] and delirium [46], we cannot formally rule out an impact of environmental light on sleep effectiveness.

Our choice to analyse only nights with at least one sleep episode longer than 10 min might have weakened our results. However, patients without sleep were not exposed to sleep-guided nursing care. Previous studies indicated that around 90% of patients fell asleep at least one time [6], suggesting that most ICU patients might benefit from sleep-guided nursing care.

REM sleep may be more difficult to detect using a single EEG electrode. However, in our experience, the REM-specific saw-tooth waves are clearly visible in ICU patients and facilitate the identification of REM sleep episodes. In addition, the REM-sleep power spectrum is markedly altered in sleep deprivation conditions with an increase in delta and theta power together with a decrease in alpha power, making REM sleep detectable using EEG spectral analysis [38]. Furthermore, our sleep scoring algorithm has been validated against polysomnography in ICU patients [26]. Finally, several sleep scoring algorithms have demonstrated excellent performance in detecting REM sleep using single EEG channel [47]. Only a small proportion of eligible patients were included in this study. However, this was a proof-of-concept study designed to demonstrate the feasibility and potential efficacy of this approach. Further studies are needed to determine which patients are most likely to benefit from this intervention.

Finally, this study was not designed or powered to assess downstream clinical outcomes such as weaning duration, delirium incidence or length of ICU stay. Our goal was to establish proof of concept that real-time sleep monitoring improves sleep continuity. Given the limited sample size and before-after design, larger and preferably randomized studies are needed to confirm these findings to determine whether these improvements translate into better clinical outcomes.

## Conclusion

Our study reports for the first time that real-time sleep monitoring can mitigate nursing intrusions when patients are asleep and thereby improve sleep effectiveness in ICUs. This innovative and efficient non-pharmacological tool promoting sleep opens exciting opportunities to test the impact of better sleep on weaning duration or delirium.

## CRediT authorship contribution statement

XD, JPF, RC and AWT drew the design; AWT, JPF, RC, AV, DC, FA, SL, LM elaborated the protocol, included the patients and made significant contribution to the paper; XD and QH acquired the data; XD, SR, QH, MAM and AWT analysed the data; XD wrote the first version; SR, FB and CR made significant contributions to the paper.

## Consent for publication

Not applicable.

## Ethics approval and consent to participate

Patients and/or their next of kin were informed and gave their consent before being included in the study. The study was approved by the independent ethics Comité de Protection des Personnes, CPP Ile de France II, number #2022-A01806-37) and registered at http://www.clinicaltrials.gov (NCT05963672).

## Funding

The University Hospital of Poitiers bought the sleep monitoring devices and paid QH’s salary to a research nurse.

## Availability of data and materials

Raw data were generated at Clinical Investigation Center INSERM 1402. Derived data supporting the findings of this study are available from the corresponding author (Xavier Drouot) on reasonable request.

## Declaration of competing interest

XD owns stock options of SomnoEngineering company, which has conceived the Sleepscan medical device used in this study to monitor sleep quality in real-time, and has a pending patent for this device. J-PF reports personal fees, non-financial support and others from Fisher&Paykel Healthcare, and personal fees and others from SOS Oxygène, outside the submitted work. AWT reports grants and personal fees from Fisher&Paykel outside the submitted work.
